# Anti-Melanogenic Effects of a Polysaccharide Isolated from *Undaria pinnatifida* Sporophyll Extracts

**DOI:** 10.3390/ijms251910624

**Published:** 2024-10-02

**Authors:** Jae-Hoon Kim, Jeong-Heon Kim, Jae-Hoon Lee, Su-Jin Eom, Nam-Hyouck Lee, Saerom Lee, Tae-Gyu Lim, Kyung-Mo Song, Min-Cheol Kang

**Affiliations:** 1 Food Processing Research Group, Korea Food Research Institute, Wanju-gun 55365, Republic of Korea; k.jaehoon@kfri.re.kr (J.-H.K.); k.jeong@kfri.re.kr (J.-H.K.); eom.su-jin@kfri.re.kr (S.-J.E.); lnh3fc@gmail.com (N.-H.L.); 2 Department of Food Science & Biotechnology, Sejong University, Seoul 05006, Republic of Korea; tglim@sejong.ac.kr; 3 Department of Food Science and Technology, Jeonbuk National University, Jeonju 54896, Republic of Korea; jokko77@gmail.com; 4 3FC Corporation, Wanju-gun 55365, Republic of Korea; 3fclsr@gmail.com; 5 Carbohydrate Bioproduct Research Center, Sejong University, Seoul 05006, Republic of Korea; 6 Department of Food Science & Biotechnology, Sungshin Women’s University, Seoul 01133, Republic of Korea

**Keywords:** *Undaria pinnatifida* sporophyll, polysaccharide, melanin, tyrosinase

## Abstract

*Undaria pinnatifida* is a temperate brown alga known to exert free radical-scavenging and anti-inflammatory effects. In this study, we investigated the skin-whitening effects of *U. pinnatifida* sporophyll extracts (UPEs) in α-melanocyte-stimulating hormone (α-MSH)-stimulated B16F10 melanoma cells. The crude polysaccharide fraction (UPF) was obtained via ethanol precipitation. Four polysaccharide fractions (UPF1–4) were isolated and purified using ion-exchange column chromatography, and their anti-melanogenic activity was evaluated. UPF3 exhibited the highest anti-melanogenic activity, showing the highest sulfate (39.79%), fucose (143 μg/mg), and galactose (208 μg/mg) contents. UPF3 significantly inhibited intracellular tyrosinase activity in B16F10 cells. We also evaluated the melanogenic signaling pathway to determine the mechanism of action of UPF3 in melanongenesis. UPF3 reduced the expression of tyrosinase-related protein-1 (TRP-1), tyrosinase-related protein-2 (TRP-2), and tyrosinase, which play important roles in melanin production. Therefore, UPF3 has high potential for use in skin-whitening functional pharmaceuticals and cosmetics.

## 1. Introduction

Melanin is a black or brown pigment present in various tissues, including hair, eyes, and skin [1]. It protects the skin from various external environmental factors, including temperature, humidity, and ultraviolet (UV) light [2]. Melanogenesis is a complex process responsible for skin pigmentation. Melanin is produced in the melanosomes of melanocytes and delivered to the keratinocytes of the epidermis [3]. Skin color is determined by the amount of melanin produced by melanocytes. Melanin production can be stimulated by UV rays and intrinsic factors such as adrenocorticotropin (ACTH), α-melanocyte-stimulating hormone (α-MSH), endothelin (EDN1), and basic fibroblast growth factor (bFGF), which increase the expression levels of microphthalmia-associated transcription factor (MITF) [4].

α-MSH stimulates the melanin production pathway. α-MSH binds to the melanocortin-1 receptor (MC1R) and is involved in the regulation of melanogenesis and pigmentation. The main intracellular signaling pathway for melanogenesis is the cyclic monophosphate/protein kinase A (cAMP/PKA) pathway, which is induced by α-MSH [5]. MITF promotes the transcription of tyrosinase-related protein-1 (TRP-1), tyrosinase-related protein-2 (TRP-2), and tyrosinase [6]. Tyrosinase is a rate-limiting enzyme that oxidizes L-tyrosine to L-3,4 dihydroxyphenylalanine (L-DOPA), which is then converted into L-dopaquinone and dopachrome [7]. TRP-2 converts dopachrome into 5,6-dihydroxyindole-2-carboxylic acid (DHICA), and TRP-1 oxidizes DHICA to melanin [8].

Melanin offers protective benefits to the skin against various environmental factors. Nevertheless, excessive melanin production can result in several skin pigmentation issues, including melasma, freckles, age spots, and hyperpigmentation disorders [9]. Consequently, the cosmetic and pharmaceutical industries are focused on tyrosinase activity inhibitors such as arbutin, kojic acid, hydroquinone, and linoleic acid to address skin pigmentation concerns [10]. However, certain chemicals can cause adverse effects, such as burns, skin irritation, and redness [11]. Thus, there is a need for the development of safe and natural whitening agents that do not carry these side effects.

*Undaria pinnatifida* is a temperate annual brown alga that is mostly found in Korea, Japan, China, the west coast of the United States, and some European countries [12,13]. *U. pinnatifida* is bitter, salty, and naturally non-toxic and has been historically used for purposes of reducing swelling and treating urological disease, thyroiditis, and gastrointestinal disease [14]. It contains a variety of bioactive compounds, including polyunsaturated fatty acids, polyphenols, peptides, vitamins, and polysaccharides [15,16]. Although *U. pinnatifida* sporophyll is a poorly utilized byproduct of *U. pinnatifida*, it contains diverse bioactive compounds, including polyphenols, polyunsaturated fatty acids, vitamins, polysaccharides, and carotenoids [17,18]. *U. pinnatifida* sporophylls are known to have antioxidant and melanogenesis-inhibitory effects [19,20]. However, the anti-melanogenic mechanisms of the polysaccharides in ultrasonic extracts have not yet been elucidated. Therefore, the aim of this study was to purify and characterize the polysaccharides in *U. pinnatifida* sporophyll extracts (UPEs) and to confirm their inhibitory effects on α-MSH-induced melanogenesis using in vitro models.

## 2. Results

### 2.1. Analysis of the Compounds in Undaria pinnatifida Sporophyll Extracts (UPEs)

**Figure 1:**
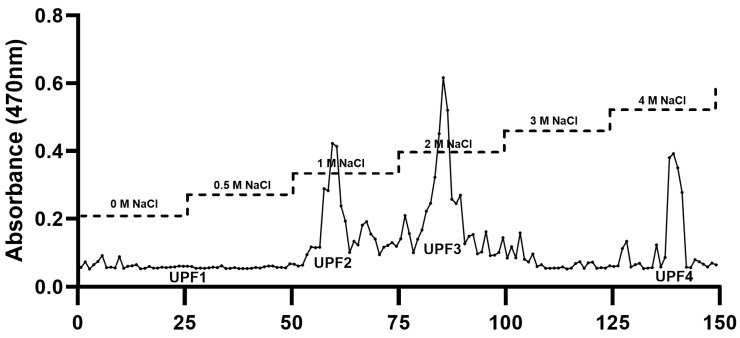
DEAE–cellulose chromatogram of polysaccharides from *Undaria pinnatifida* sporophyll crude polysaccharides.

Crude polysaccharides from UPEs were isolated by adding ethanol to the UPEs, followed by the separation of the polysaccharides through anion-exchange chromatography using a DEAE cellulose column with a NaCl gradient ranging from 0 to 4 M. The chromatogram was divided into four fractions, which were quantified by the phenol–sulfuric acid colorimetric method utilizing the polysaccharide fractions from the anion-exchange column. The fractions in the chromatogram were indicated by four distinct peaks: UPF1, UPF2, UPF3, and UPF4 (Figure 1).

Table 1 shows that the UPEs were predominantly composed of polysaccharides and sulfates. In particular, the UPF, which was prepared by ethanol precipitation, contained high levels of polysaccharides and sulfates. UPF3 of the DEAE fractions also contained high levels of polysaccharides and sulfates. The monosaccharide compositions of the UPEs listed in Table 2 show that the UPEs comprised four monosaccharides: fucose, rhamnose, galactose, and glucose. In the UPEs, galactose was the predominant monosaccharide, constituting 49.5% (82 μg/mg of dry weight extract) of the total polysaccharide content, with fucose following at 41.65% (70 μg/mg of dry weight extract). Among the fractions, UPF3 had the highest levels of fucose (143 μg/mg of dry weight extract) and galactose (208 μg/mg of dry weight extract).

### 2.2. Effects of the UPEs on Cytotoxcity in B16F10 Cells

**Figure 2:**
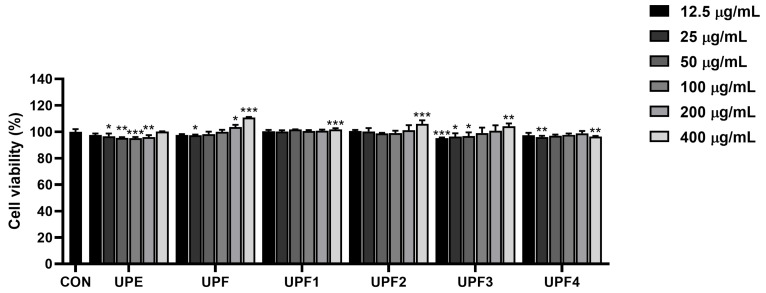
Cytotoxicity of UPEs on B16F10 cells. * *p* < 0.05, ** *p* < 0.01, and *** *p* < 0.001 compared to the untreated control group.

The cytotoxicity of the samples was evaluated using B16F10 cells. The cells were treated with fractionated samples at concentrations of 12.5, 25, 50, 100, 200, and 400 μg/mL to examine cytotoxicity. The cytotoxicity of the sample-treated group was not significantly lower than that of the untreated group. These results indicated that the samples were not cytotoxic to B16F10 cells up to a concentration of 400 μg/mL (Figure 2). Therefore, the experiments were conducted at non-toxic concentrations of 100, 200, and 400 μg/mL.

### 2.3. Effects of the Samples’ Melanin Contents and Tyrosinase Activity in B16F10 Cells

**Figure 3:**
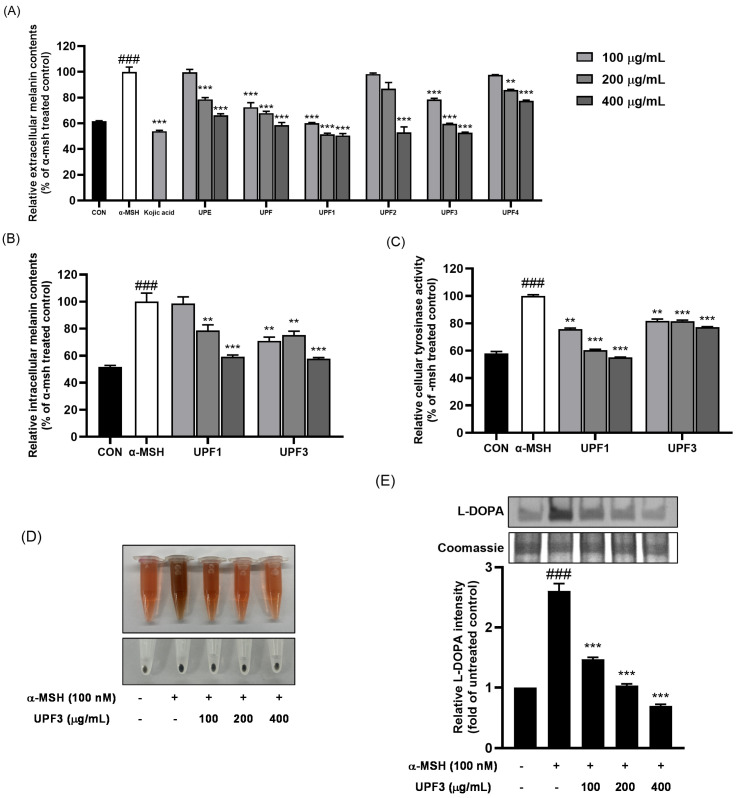
Melanogenic effects of UPEs on B16F10 cells. (**A**) Extracellular melanin contents of UPEs. (**B**) Intracellular melanin contents of UPF1 and UPF3. (**C**) Cellular tyrosinase activity of UPF1 and UPF3. (**D**) Extracellular melanin content image of UPF3. (**E**) Cellular tyrosinase activity determined by L-DOPA staining assay. ^###^ *p* < 0.001 compared to the untreated control group. ** *p* < 0.01, and *** *p* < 0.001 compared to the α-MSH-treated group.

The anti-melanogenic effects of the fractionated samples were assessed by measuring the extracellular melanin content in α-MSH-stimulated B16F10 cells (Figure 3A). Compared with those in the α-MSH-stimulated group, the extracellular melanin contents of the UPF, UPF1, and UPF3 were significantly reduced in a dose-dependent manner compared to those in the positive group examining kojic acid. To confirm this, the intracellular melanin contents and cellular tyrosinase enzyme activity of UPF1 and UPF3 were evaluated (Figure 3B,C). These results indicated that UPF3 was a significantly effective fraction with anti-melanogenic effects in B16F10 cells (Figure 3D). The anti-melanogenic effect of UPF3 was confirmed by L-DOPA staining. The reduction in tyrosinase enzyme activity following UPF3 treatment in B16F10 cells was confirmed by the weak band intensity compared to that observed in the α-MSH-stimulated cells (Figure 3E).

### 2.4. Effects of UPF3 on the Expression of Melanogenic Proteins in B16F10 Cells

**Figure 4:**
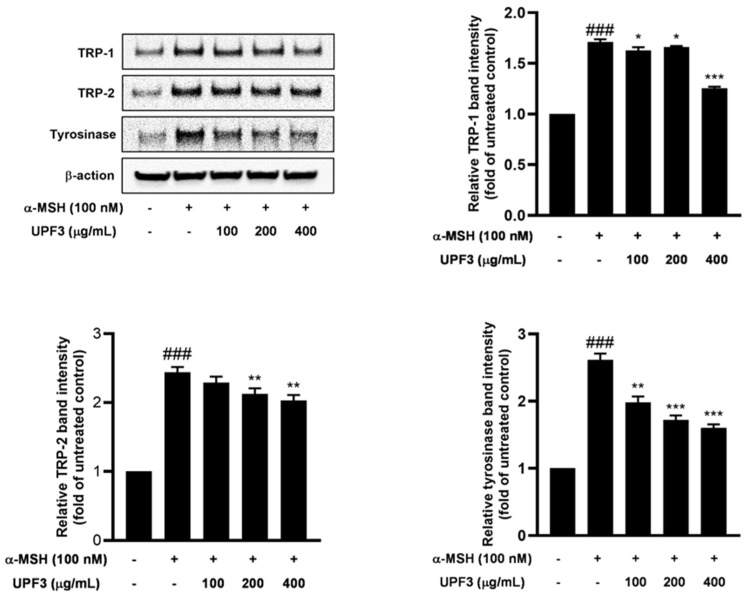
Effects of UPF3 on melanogenesis-related signaling pathways as assessed by Western blot in B16F10 cells. ^###^ *p* < 0.001 compared to the untreated control group. * *p* < 0.05, ** *p* < 0.01, and *** *p* < 0.001 compared to the α-MSH-treated group.

To validate the downregulatory effect of UPF3 treatment on melanogenic enzymes, we compared the protein expression of tyrosinase, TRP-1, and TRP-2 in UPF3-treated B16F10 cells using Western blotting. Their expression levels were significantly upregulated in α-MSH-treated cells compared to the untreated group. Figure 4 shows that UPF3 treatment significantly reduced the protein expression of key enzymes involved in melanogenesis (tyrosinase, TRP-1, and TRP-2). These results demonstrated that UPF3 inhibits cellular melanogenesis in cells stimulated by α-MSH by downregulating the expression of melanogenic proteins in a dose-dependent manner.

## 3. Discussion

Melanin is the pigment responsible for protecting the skin from various external environmental factors [2]. However, excessive melanin production can cause several skin pigmentation disorders, including freckles and hyperpigmentation [9]. This study focused on developing a non-toxic and natural melanogenic inhibitor using UPEs.

*Undaria pinnatifida* sporophylls were extracted using ultrasonic extraction, as per our previous research [20]. The extracts were precipitated using ethanol precipitation, followed by purification through anion-exchange chromatography using a DEAE cellulose column. The separated samples were divided into four fractions by measuring their polysaccharide contents using the phenol–sulfuric acid method. This resulted in extracts (UPEs), a precipitate fraction (UPF), and four separated fractions (UPF1–4).

In this study, we first analyzed the monosaccharide contents and chemical compositions of the UPEs. UPEs contain high amounts of galactose and fucose, with UPF3 having the highest fucose (143 μg/mg of dry weight extract, 36.49%) and galactose (208 μg/mg of dry weight extract, 58.38%) contents. These contents are very high compared to the 6~10% fucose and 15~25% galactose contents observed in a previous study showing that these monosaccharides have good whitening effects [21]. Galactose inhibits melanogenesis by regulating the cAMP/PKA/Akt signaling pathways [22]. Among the UPEs, UPF3 exhibited the highest sulfate content. Fucoidan, a marine sulfated biopolysaccharide with a heterogeneous and complex chemical structure, is known to be a fucose-containing sulfated polysaccharide [23]. Since UPF3 is high in fucose and sulfates, it contains large amounts of fucoidan. Fucoidan has been reported to inhibit melanin production by regulating the ERK/CREB/MITF pathways [24,25]. The anti-melanogenic effects of the UPEs were evaluated by measuring intracellular melanin contents and tyrosinase enzyme activity, which confirmed the efficiency of UPF1 and UPF3 in reducing extracellular melanin contents. We found that UPF3 had a powerful inhibitory effect on melanin synthesis and also showed the inhibition of tyrosinase enzyme activity, as demonstrated by tyrosinase zymography. Therefore, the melanin-inhibitory activity of UPF3 was likely due to its high galactose and fucoidan contents.

α-MSH, an intrinsic factor in the intracellular melanin production pathway, was applied to B16F10 cells to stimulate melanogenesis. α-MSH released from UV-exposed keratinocytes activates the cAMP/PKA/CREB axis, stimulating melanin biosynthesis [26]. Activated MITF increases the expression of TRP-1, TRP-2, and tyrosinase, leading to intracellular pigmentation [27]. In this study, 100 nM α-MSH was applied to B16F10 cells as an intracellular melanogenesis stimulator, along with different concentrations of UPEs, for 72 h. Figure 3 demonstrates that the UPEs inhibited melanogenesis, with UPF1 and UPF3 being particularly effective. Upon observing the intracellular responses of the cells to UPF3 treatment, the increased conversion of L-DOPA into dopachrome stimulated by α-MSH during melanogenesis was found to be successfully decreased. UPF3 suppressed tyrosinase enzyme activity and significantly reduced dopachrome production from L-DOPA. Figure 4 shows the effects of UPF3 on the expression of the melanogenic enzymes TRP-1, TRP-2, and tyrosinase, revealing that UPF3 effectively inhibited melanin synthesis in B16F10 cells stimulated by α-MSH.

Melanin synthesis is a complex process involving several molecular biological factors [5,6]. MITF is a pivotal factor that regulates the transcription of melanin synthesis enzymes and is involved in the cAMP/PKA/CREB cascade [28]. α-MSH promotes the production of cAMP, which induces phosphorylation of the CREB transcription factor, in turn promoting MITF activation. This series of events upregulates the transcription of the melanin synthesis enzymes TRP-1, TPR-2, and tyrosinase [29,30]. Therefore, we hypothesized that UPF3 regulates the cAMP/PKA/CREB pathways to decrease the expression of the key melanogenic enzymes TRP-1, TRP-2, and tyrosinase, thereby inhibiting melanin synthesis.

## 4. Materials and Methods

### 4.1. Materials and Reagents

DEAE Sepharose Fast Flow was purchased from Cytiva (Marlborough, MA, USA). α-MSH, L-DOPA, Folin–Ciocalteu reagent, and RIPA lysis buffer were obtained from Sigma-Aldrich (St. Louis, MO, USA). A Pierce BCA Protein Assay Kit, a Slide-A-Lyzer^TM^ Dialysis Flask, and 3-(4,5-Dimethylthiazol-2-yl)-2,5-Diphenyltetrazolium Bromide (MTT) were purchased from Thermo Fisher Scientific (Waltham, MA, USA). A Protease/Phosphatase Inhibitor Cocktail and a primary antibody against β-actin were purchased from Cell Signaling Technology (Danvers, MA, USA). Primary antibodies against tyrosinase, TRP-1, and TRP-2 were purchased from Abcam (Cambridge, UK).

### 4.2. Ultrasonic Extraction of Undaria pinnatifida Sporophylls

*U. pinnatifida* sporophylls were obtained from a local market in Wando (Jeollanamdo, Republic of Korea). The *U. pinnatifida* sporophylls were dried and powdered using a blender. The powdered samples (400 g) were mixed with water (40 L). The samples were extracted using MX Sonic (MX-12S2, Mirae Ultrasonic Tech., Bucheon, Republic of Korea) at 1080 W, 80% amplitude, 20 kHz, and 30 °C for 8 h. After extraction, the extracts were centrifuged (3000× *g*, 30 min, and 4 °C), and the *Undaria pinnatifida* sporophyll extracts (UPEs) were lyophilized.

### 4.3. Crude Polysaccharide Separation

The UPEs in DW were mixed well with twice the volume of ethanol. Then, the mixture was stored overnight at 4 °C. The crude polysaccharide separation fraction (UPF) was collected by centrifugation (12,000× *g*, 30 min, and 4 °C) [31].

### 4.4. Anion-Exchange Chromatography (DEAE)

The UPF obtained using the aforementioned procedure was subjected to a DEAE cellulose column (40 × 3 cm), equilibrated with 50 mM sodium acetate, and washed with 50 mM NaCl. Elution was carried out at a flow rate of 2 mL/min using a linear gradient of 0 M to 4 M NaCl. Fractions (10 mL each) were collected and analyzed for their polysaccharide contents using a phenol–H_2_SO_4_ assay. These fractions were then dialyzed and freeze-dried to yield UPF1, UPF2, UPF3, and UPF4.

### 4.5. Polysaccharide, Protein, Polyphenol, and Sulfate Content Analyses

The polysaccharide concentrations of the UPEs were quantified using the phenol–sulfuric acid assay [32]. The samples were dissolved in DW, transferred into tubes, and mixed with 5% phenol solution. Subsequently, H_2_SO_4_ was added, and the mixture was cooled to room temperature. The absorbance of the final solution was measured at 470 nm. The total polysaccharide content was then calculated using glucose as the reference standard.

The protein contents of the UPEs were determined using the Pierce BCA Protein Assay Kit. The samples were mixed with the BCA reagent and shaken for 30 min. The absorbance of the mixture was measured at 562 nm. The total protein content was calculated using Bovine Serum Albumin (BSA) as the standard.

The polyphenol contents of the UPEs were determined using the Folin–Ciocalteu reagent [33]. The samples were mixed with the Folin–Ciocalteu reagent (1N), and 20% Na_2_CO_3_ was added to the wells. After 30 min, absorbance was measured at 700 nm. The total polyphenol content was calculated using gallic acid as the standard.

The sulfate concentrations of the UPEs were analyzed using the barium chloride gelatin assay [34]. The samples were combined with HCl and heated in a drying oven at 105 °C for 5 h. Afterward, the samples were treated with a 6.25% HNO_3_ and Gum–Arabic–HAc–BaCl_2_ mixture and left to react for 10 min in the dark. The absorbance was recorded at 440 nm, and the sulfate content was determined using (NH_4_)_2_SO_4_ as the reference standard.

### 4.6. Analysis of Molecular Weights and Monosaccharide Compositions through High-Performance Liquid Chromatography

Size exclusion chromatography was employed to determine the molecular weights of the UPEs [35]. All samples (5 mg/mL in deionized water) were filtered through a 0.45 μm syringe filter, and the filtered samples were injected into a column (protein KW-803; 8 mm × 300 mm, 4 μm; Shodex, Tokyo, Japan). The injection volume was 10 μL, and the flow rate was maintained at 0.8 mL/min. Peaks were detected using a JASCO HPLC system equipped with a refractive index (RI) detector (RI-2031 Plus) with a runtime of 25 min per injection. The molecular weight was determined using pullulan standards (Shodex Standard P-82; Showa Denko, Tokyo, Japan).

For the analysis of monosaccharide compositions, high-performance anion-exchange chromatography with pulsed amperometric detection (Dionex, Sunnyvale, CA, USA) was employed [36]. A CarboPac^TM^ PA1 column (2 mm × 250 mm, 10 μm particle size) was utilized for the separation process. Each sample (2 mg/mL) was hydrolyzed using trifluoroacetic acid before being injected into the column. The injection volume was set at 20 μL, and the eluent (18 mM NaOH/200 mM NaOH) was run at a flow rate of 1.0 mL/min.

### 4.7. Cell Culture and Viability Assays

B16F10 mouse melanoma cells were purchased from the American Type Culture Collection (ATCC, Rockville, MD, USA). The B16F10 cells were cultured in Dulbecco’s modified Eagle’s medium (DMEM), supplemented with 10% fetal bovine serum (FBS) and 1% penicillin/streptomycin (P/S). The cells were incubated at 37 °C in a humidified atmosphere containing 95% air and 5% CO_2_. Cell viability was analyzed using a 3-(4,5-Dimethylthiazol-2-yl)-2,5-Diphenyltetrazolium Bromide (MTT) assay. The cells were seeded in a 96-well plate at 1 × 10^4^ cells/well and incubated for 24 h. The cells were then treated with UPEs at various concentrations (12.5, 25, 50, 100, 200, and 400 μg/mL) for 24 h. After the treatment, the cells were incubated with MTT solution for 2 h. Formazan crystals were then dissolved in DMSO, and the absorbance was measured at 540 nm.

### 4.8. Melanin Content Assay

B16F10 cells were seeded in a 60 mm culture dish at a density of 1 × 10^5^ cells/dish. After 24 h of incubation, the cells were pretreated with UPEs (100, 200, and 400 μg/mL) for 1 h and then treated with α-MSH (100 nM) for 72 h. Extracellular melanin contents were measured in the culture media by assessing the absorbance at 490 nm. The cells were washed twice with DPBS and incubated with 1 M NaOH solution to dissolve intracellular melanin. Dissolved melanin was quantified by measuring the absorbance at 490 nm using a microplate reader.

### 4.9. Cellular Tyrosinase Assay

B16F10 cells were seeded in a 60 mm culture dish at a density of 1 × 10^5^ cells/dish and incubated for 24 h. The cells were pretreated with UPEs (100, 200, and 400 μg/mL) for 1 h, followed by treatment with α-MSH (100 nM) for 72 h. Afterward, the cells were harvested and lysed using RIPA lysis buffer and then centrifuged at 12,000× *g* for 10 min, and the supernatants were collected. The protein content in the lysate was determined using the Pierce BCA Protein Assay Kit. Then, 40 μL of the lysate (30 μg) and 100 μL of L-DOPA (10 mM) were mixed in a 96-well plate and incubated at 37 °C for 1 h. Cellular tyrosinase activity was assessed by measuring dopachrome absorbance at 475 nm.

### 4.10. L-DOPA Staining

The L-DOPA staining assay was conducted as previously described [37]. B16F10 cells were seeded in a 60 mm culture dish at a density of 3 × 10^5^ cells/dish and incubated for 24 h. The cells were pretreated with UPEs (100, 200, and 400 μg/mL) for 1 h, followed by treatment with α-MSH (100 nM) for 72 h. The cells were then washed twice with DPBS, lysed with RIPA lysis buffer, and centrifuged at 12,000× *g* for 10 min, after which the supernatant was collected. The protein concentration in the lysate was determined using the Pierce BCA Protein Assay Kit. The proteins (30 μg) were combined with loading buffer without β-mercaptoethanol. Then, the proteins were separated on a 10% SDS gel and washed three times with 0.1 M sodium phosphate monobasic buffer for 30 min. The gel was subsequently immersed in a staining solution containing 0.1 M sodium phosphate monobasic buffer containing 10 mM L-DOPA and incubated in the dark at 37 °C for 2 h. After staining, tyrosinase activity was detected as dark melanin-containing bands on the gel through PrintGraph 2M (ATTO, Tokyo, Japan).

### 4.11. Western Blotting Analysis

B16F10 cells (3 × 10^5^ cells/dish) were seeded in a 60 mm dish and incubated for 24 h. The cells were pretreated with UPEs (100, 200, and 400 μg/mL) for 1 h and then treated with α-MSH (100 nM). After 24 h, proteins were extracted using RIPA lysis buffer supplemented with a Protease/Phosphatase Inhibitor Cocktail. The protein concentration of the lysate was assessed using the Pierce BCA Protein Assay Kit. The extracted proteins were separated on 10% SDS acrylamide gels and transferred onto polyvinylidene difluoride (PVDF) membranes. The membranes were blocked with EveryBlot Blocking buffer (Bio-Rad, Hercules CA, USA) for 15 min at room temperature followed by overnight incubation with primary antibodies (β-actin: 4970s; tyrosinase: ab1709015; TRP-1: ab1708676; and TRP-2: ab74073) at 4 °C. After washing with TBST several times, the membranes were incubated with secondary antibodies (anti-rabbit IgG and HRP-linked antibody: 7074s; anti-mouse IgG and HRP-linked antibody: 7076s) at room temperature for 1 h. Protein expression was visualized using an ECL reagent and observed using a chemiluminescence reader (EZ-Capture MG, ATTO, Tokyo, Japan).

### 4.12. Statistical Analysis

All the experiments were repeated at least three times, and the results are presented as the mean ± standard deviation. Normality tests were carried out to assess whether the data followed a normal distribution. For data that were normally distributed, statistical significance was assessed using Student’s *t*-test with the SPSS program 22.0 (Chicago, IL, USA), and significant differences were considered at *p* < 0.05. Pearson’s correlation analysis was also performed using SPSS, with the significance level set at *p* < 0.01.

## 5. Conclusions

The UPEs extracted from *U. pinnatifida* sporophylls by ultrasonication exhibited anti-melanogenic activity. In particular, UPF3, which was isolated by anion-exchange chromatography, demonstrated potent anti-melanogenic effects on α-MSH-stimulated B16F10 melanoma cells by inhibiting tyrosinase signaling pathways and enzyme activity. The anti-melanogenic effects of UPF3 were attributed to its high galactose and fucoidan contents; however, further experiments, such as skin organoid cell, 3D cell culture, and human skin experiments, are needed to substantiate its whitening effects more conclusively. These findings strongly suggest that UPF3 is a promising natural skin-whitening agent for the prevention of pigmentary disorders.

## Figures and Tables

**Table 1:** Chemical composition analysis.

Sample	Chemical Composition (%)			
	Monosaccharides	Proteins	Total Polyphenols	Sulfates
UPE	15.17 ± 0.44	4.39 ± 0.17	0.12 ± 0.00	25.02 ± 0.41
UPF	35.36 ± 1.42	7.92 ± 0.24	0.32 ± 0.01	29.56 ± 0.1
UPF1	12.69 ± 1.17	1.35 ± 0.12	nd	0.93 ± 0.12
UPF2	17.07 ± 2.94	0.47 ± 0.22	nd	1.24 ± 0.08
UPF3	29.46 ± 2.94	1.87 ± 0.1	nd	39.79 ± 0.21
UPF4	3.99 ± 0.8	1.83 ± 0.55	0.05 ± 0.00	1.14 ± 0.05

nd: not determined.

**Table 2:** Monosaccharide composition analysis.

		UPE	UPF	UPF1	UPF2	UPF3	UPF4
Monosaccharide composition (μg/mg of dry weight extract (%))	Fucose	70 (41.65)	61 (37.09)	6 (7.62)	8 (26.68)	143 (36.49)	11 (22.73)
	Rhamnose	2 (0.67)	2 (0.58)	7 (7.27)	1 (2.51)	3 (0.61)	0 (0.45)
	Arabinose	nd	nd	nd	nd	nd	nd
	Galactose	82 (49.5)	83 (50.88)	23 (32.18)	1 (3.54)	208 (58.38)	17 (36.76)
	Glucose	5 (6.54)	9 (3.52)	21 (20.72)	9 (35.49)	2 (0.32)	5 (10.69)
	N/A	5 (6.54)	7 (7.93)	13 (20.72)	7 (30.04)	12 (4)	5 (13.19)

nd: not determined. N/A: not available.

## Data Availability

All data are contained within the article.
